# The attenuated *Mycoplasma bovis* strain promotes apoptosis of bovine macrophages by upregulation of CHOP expression

**DOI:** 10.3389/fmicb.2022.925209

**Published:** 2022-08-03

**Authors:** Hui Zhang, Siyi Lu, Jin Chao, Doukun Lu, Gang Zhao, Yingyu Chen, Huanchun Chen, Muhammad Faisal, Liguo Yang, Changmin Hu, Aizhen Guo

**Affiliations:** ^1^State Key Laboratory of Agricultural Microbiology, College of Veterinary Medicine, Huazhong Agricultural University, Wuhan, China; ^2^College of Animal Husbandry and Veterinary Medicine, Southwest Minzu University, Chengdu, China; ^3^College of Animal Science and Technology, Huazhong Agricultural University, Wuhan, China; ^4^Key Laboratory of Development of Veterinary Diagnostic Products, Huazhong Agricultural University, Wuhan, China; ^5^Hubei Hongshan Laboratory, Huazhong Agricultural University, Wuhan, China

**Keywords:** *Mycoplasma bovis*, CHOP, attenuation, apoptosis, transcriptome, endoplasmic reticulum stress, intracellular survival

## Abstract

*Mycoplasma bovis (M. bovis)* is one of the major pathogens in the bovine respiratory disease complex, which includes pneumonia, mastitis, and arthritis and causes a great economic loss in the cattle industry. In China, a live-attenuated vaccine strain *M. bovis* P150 was obtained by a continuous culture of the wild-type strain *M. bovis* HB0801 (P1) *in vitro* for 150 passages. Using the infected bovine macrophage cell line BoMac, this work attempted to investigate the mechanism of P150 attenuation and protective immune response. To begin, we show that *M. bovis* P150 effectively triggered cytotoxicity and apoptosis in BoMac, although with lower intracellular survival than P1. The transcriptomes of BoMac after infection with *M. bovis* strains P1 and P150 were sequenced, and bioinformatic analysis identified 233 differentially expressed genes (DEGs), with 185 upregulated and 48 downregulated. Further Gene Ontology (GO) and Kyoto encyclopedia of genes and genomes (KEGG) pathway enrichment analyses revealed that the majority of the DEGs were linked to CHOP complex, MAP kinase phosphatase activity and were involved in the IL-17 signaling pathway in immune response, MAPK signaling pathway in signal transduction, and p53 signaling pathway in cell growth and death. Among them, the level of C/EBP homologous protein (CHOP) was significantly upregulated in P150-infected BoMac compared to P1-infected cells at different time points, along with its upstream and downstream genes phosphorylated-PERK, phosphorylated-EIF2α, ATF4, and GADD45A increased in the PERK-dependent ER stress response. The role of CHOP in apoptosis was further verified by *M. bovis-*induced siCHOP knockdown in BoMac cells. The results showed that CHOP knockdown enhanced P150-induced apoptosis and dramatically increased the *M. bovis* P1 and P150 intracellular survival, particularly for P150. These data suggest that P150 infection upregulates CHOP expression, which can increase apoptosis and mediate a crosstalk between ER stress and apoptosis during infection, and hence, contribute to high cytotoxicity and low intracellular survival.

## Introduction

*Mycoplasmas* are the most basic self-replicating prokaryotes that are found in both humans and animals. They colonize the mucous surface of the respiratory, urogenital tracts, mammary glands, eyes and joints, exhibiting organ and tissue specificity (Citti and Blanchard, 2013). *Mycoplasma bovis* (*M. bovis*) is a common pathogen in cattle causing bovine mycoplasmosis, which can cause pneumonia, mastitis, genital disorders, arthritis, otitis, keratoconjunctivitis, or meningitis (Pfützner and Sachse, 1996; Alberti et al., 2006; Maunsell et al., 2012; Suwanruengsri et al., 2021). These illnesses have significant economic consequences, resulting in losses in the beef and dairy industries around the world (Calcutt et al., 2018). Due to the lack of effective vaccine, a restricted drug repertoire for disease treatment and increased development of antimicrobial resistance of *M. bovis*, management of bovine mycoplasmosis has remained difficult (Dudek et al., 2021).

Inactivated vaccines elicited a modest immunological response, which was insufficient to create antibodies against reinfection. For example, inactivated *Brucella* vaccines have been found to have issues such as inadequate protection, local responses caused by adjuvants, and serological issues (Lalsiamthara and Lee, 2017). Bacille Calmette-Guerin (BCG) is the only licensed vaccination against *Mycobacterium tuberculosis*, and it is more effective than inactivated vaccine (Fatima et al., 2020). It was observed that inactivated vaccines for enzootic pneumonia in mycoplasma species have improved, but illness decrease has been minimal (Nicholas et al., 2009). So far, a recent study shows that live-attenuated vaccines for several mycoplasmosis have already been created and have a higher effectiveness, such as *Mycoplasma gallisepticum* strain ts-304 (Kanci Condello et al., 2020) and *Mycoplasma synoviae* strain MS-H (Zhu et al., 2019) through chemical mutagenesis, and *Mycoplasma hyopneumoniae* strain 168 through *in vitro* continuous passaging (Xiong et al., 2014). After continuous development, *in vitro* for 150 passages, we generated an attenuated vaccine *M. bovis* strain P150 from the wild-type *M. bovis* HB0801 strain (designated as P1) after continuous culture *in vitro* for 150 passages (Zhang et al., 2014). The P150 strain has low virulence but remains a critical protection against a virulent challenge from the P1 strain (Chao et al., 2019). Further genome sequencing indicated that P150 genome was missing one 14.2-kb section and that the strain has 46 non-sense single-nucleotide polymorphisms and indels (Rasheed et al., 2017). The P1 infection tended to induce a pro-inflammatory pathogenic Th17 response in bovine peripheral blood mononuclear cells, whereas P150 infection induced a Th1 response (Chao et al., 2019). However, the mechanism of the P150 strain’s attenuation and protective immune response in comparison to the P1 strain is unknown.

Through successful recognition, clearance of germs, cytokine generation, and antigen presentation, macrophages play a crucial role in the early management of bacterial infections. Variation in surface antigens, production of biofilms and cleavage of the IgG heavy chains are only a few of the ways *M. bovis* uses to avoid opsonization and phagocytosis by the macrophages, allowing it to avoid the activation of an effective immune response (Maunsell and Chase, 2019). To limit bacterial infection, host macrophages also send out an apoptotic signal (Borchsenius et al., 2020). *M. bovis* has been shown to delay apoptosis in bovine monocytes *in vitro*, extending bacterial viability and facilitating bacterial dissemination (Mulongo et al., 2014). *M. bovis* may also suppress the intrinsic apoptotic pathway in bovine macrophage BoMac cells (Maina et al., 2019). However, *M. bovis* of varying virulence has been observed to modulate PBMC proliferation, apoptosis, and survival in distinct ways (Suleman et al., 2016). Understanding how *M. bovis* infection causes apoptosis would aid in identifying host immune responses that could be used to limit infection.

This study aimed to investigate the mechanism by which P150 was attenuated by comparing the transcriptome sequences of BoMac cells infected with *M. bovis* P1 and P150, and revealing the function of critical genes associated with the viability of infected BoMac cells and mycoplasma survival after infection. The findings suggest that elevation of CHOP expression may be responsible for the increased apoptosis produced by P150. The findings add to our understanding of *M. bovis* P150 attenuation and likely explain the protective immune response.

## Materials and methods

### Strains, cells, and reagents

*Mycoplasma bovis* HB0801 wild-type strain (P1) (GenBank accession no. NC_018077.1) was isolated from the lung of a diseased cattle with pneumonia in Hubei Province, China (Qi et al., 2012), and its attenuated strain *M. bovis* P150 strain (P150) (GenBank accession no. CP007590.1) was produced from P1 following continuous passaging *in vitro* 150 times and maintained in our laboratory (Zhang et al., 2014). Both strains were cultivated at 37°C in 5% CO_2_ for 36 h in pleuropneumonia-like organisms (PPLO) medium (BD Company, MD, United States) supplemented with 10% horse serum (Hyclone, UT, United States). A 0.1 ml of culture with serial dilutions was plated on the PPLO medium containing 1.5% agarose (BD Difco™, San Diego, United States) and incubated at 37°C in 5% CO_2_ for 72 h to count the quantity of mycoplasma (CFU/ml).

BoMac, a bovine peritoneal macrophage cell line donated by Judith R. Stabel of the United States Department of Agriculture’s Johne’s disease Research Project in Ames, Iowa, was grown in Roswell Park Memorial Institute (RPMI) medium supplemented with 10% heat-inactivated fetal bovine serum (FBS) (Gibco, NY, United States).

The Cell Signaling Technology company (Danvers, MA, United States) provided antibodies against PERK Rabbit mAb (5683), Phospho-PERK Rabbit mAb (3179), phosphor-eIF2α Rabbit mAb (3398), CHOP Mouse mAb (2895), GADD45A Rabbit mAb (4632). Proteintech (Wuhan, China) supplied the β-actin Mouse mAb (60008-1). All these prime antibodies have species cross-reactivity with bovine cells according to the product manual. The secondary antibodies used HRP-conjugated goat anti-mouse or anti-rabbit IgG (SouthernBiotech Company, Birmingham, United Kingdom).

### Cell viability assay

The MTT assay was used to investigate the *M. bovis* P1 and P150 on the viability of BoMac cells. BoMac cells (1 × 10^4^ cells/100 μl) were plated overnight in a 96-well plate and infected with *M. bovis* P1 and P150 at different multiplicities of infection (MOI) of 0.1, 1, 10, 100, 500, 1000 for another 12 h at 37°C. Each well received a 10 μl MTT solution (Beyotime Biotechnology, Shanghai, China) and was incubated for additional 4 h. Then, on a shaker at a low speed for 10 min, 110 μl of formazan solution was added to each well to fully dissolve the crystals. The absorbance at 490 nm was measured using a microplate reader (Victor NIVO, PerkinElmer Inc., United States). The cell viability was determined using the formula: cell viability (%) = (OD_sample_-OD_blank_)/(OD_nc_-OD_blank_) × 100%.

### Cell apoptosis assay

*Mycoplasma bovis* P1 and P150 strains were infected into BoMac cells (1 × 10^6^ cells/ml) at various MOIs of 10, 100, 500, 1,000, and 2,000 for 12 h, or at an MOI of 1,000 for 3, 6, 12, and 24 h. The cells were collected and stained with the Annexin V-FITC/PI detection kit (Vazyme, Nanjing, China) according to the manufacturer’s protocol for flow cytometry detection, and the apoptotic cells were detected using a flow cytometer (Cytoflex, Beckman Coulter, Inc., United States) and analyzed using the FlowJo VX software.

### *Mycoplasma bovis* survival in BoMac by gentamicin protection assay

The gentamicin assay was used to assess mycoplasma survival in BoMac cells, as previously described (Burgi et al., 2018). *M. bovis* P1 and P150 were infected into BoMac cells at MOI of 50. The cells were rinsed twice with PBS after 3 h of infection, then given the DMEM medium supplemented with 400 μg/ml gentamicin. The plates were incubated for another 3 h before being washed three times with PBS to remove gentamicin and replaced with fresh DMEM medium. The cells were then collected at 0, 3, 12, 24, 36, and 50 h, lysed with sterile water, and the amount of mycoplasma (CFU/ml) was determined by plating the 10-fold serially diluted lysate on PPLO agar plates.

### Observation of cell morphology and ultrastructure with transmission electronic microscopy

*Mycoplasma bovis* and the infected cells were seen using transmission electron microscopy (TEM) (HITACHI, Tokyo, Japan). BoMac cells were seeded in 6-well plates, infected at an MOI of 1,000 with *M. bovis* P1 and P150, and incubated for 12 h. The cells were then rinsed in PBS and fixed with 1.5% glutaraldehyde in 0.1 M cacodylate buffer, then postfixed for 2 h at 4°C in 2% osmium tetroxide, dehydrated in a graded series of ethanol, embedded in Epon 812, and cut into ultrathin section (75 nm). Before being examined with a HITACHI H-600 electron microscope at 80 kV, the slices were stained with uranyl acetate and lead citrate.

### RNA extraction, RNA sequencing, and bioinformatic analysis

BoMac cells (1 × 10^6^ cells/ml) were seeded into 6-well cell plates and inoculated with *M. bovis* P1 and P150 at an MOI of 1,000 for 12 h. The entire RNA extraction, cDNA library construction, and RNA-seq method were carried out as previously described (Ouyang et al., 2016). Total RNA was isolated using TRIzol^®^ Reagent (Invitrogen, United States), quality-verified using agarose gel electrophoresis, and quantified using an Agilent 2100 bioanalyzer (Agilent Technologies, CA, United States). The RNA integrity numbers (RIN) greater than eight were further processed for sequencing.

The mRNA was extracted using magnetic oligo (dT) beads and cleaved into short fragments after total RNA samples were treated with DNase I. Then, using the mRNA fragments as templates, cDNA was generated and appropriate fragments purified by agarose gel electrophoresis were chosen as PCR templates. Finally, the Illumina HiSeq high-throughput sequencing platform was used to sequence six samples from each of the three groups. After filtering the adapters, clean reads were recovered and mapped to reference sequences using HISAT2. The DEseq2 program was then used to find differentially expressed genes (DEGs) in P1 and P150 infected cells. The significance of gene expression differences was judged using the *p* < 0.05 and the fold change (log_2_ratio > 1) thresholds. The Clustering software was used to do hierarchical cluster analysis of DEGs.

### Functional categorization

The generated list of DEGs was analyzed using DAVID^1^ and KOBAS^2^ online tools to perform Gene Ontology (GO) enrichment analysis and KEGG pathway enrichment analysis, respectively.

### Validation of sequencing data by qRT-PCR assay

A total of fourteen DEGs with substantial fold changes at a false discovery rate of *p* < 0.01 were chosen for qRT-PCR validation of expression diversity to validate the RNA-seq results. As previously stated, the total RNA was isolated from BoMac cells using TRIzol (Invitrogen, Carlsbad, California, United States). cDNA was synthesized using 1 μg of total RNA and a reverse transcription (RT) kit (Vazyme, Nanjing, China). Further, qRT-PCR was performed using the SYBR Green master mix (Vazyme). Each target gene’s expression was compared to that of GAPDH. Table 1 lists the primer sequences designed by us that were used in this study.

**TABLE 1:** The primers used for qRT-PCR in this study.

Primers	Sequences (5′-3′)	Amplicon size (bp)
CXCR4-F	GCAGGTAGCAAAGTGACCCT	162
CXCR4-R	CGGAAGCAGGGTTCCTTCAT	
CHAC1-F	CAACCACTCAAGGCATTGGC	130
CHAC1-R	AGTACTCAAGGTTGTGCCCG	
CXCL8-F	ATTCCACACCTTTCCACCCC	126
CXCL8-R	CCTTGGGGTTTAGGCAGACC	
GADD45A-F	CTCGGCTGGAGAGCAAAAGA	235
GADD45A-R	CTCACAGCAGAATGCCTGGA	
DDIT4-F	GCAAAGAACTACTGCGCCTG	206
DDIT4-R	GGCAGAGCTAAACAACCCCT	
PPP1R15A-F	CAACCAGGAGACACAGAGGA	222
PPP1R15A-R	ACTCTGGGTCTGAAGGGAGG	
CHOP-F	CCTGAGGAGAGAGTGTTCCAG	219
CHOP-R	CTCCTTGTTTCCAGGGGGTG	
TCIM-F	GTAAGACCCTGACACGCACA	206
TCIM-R	TGACATCAGCGCCAGTCTTT	
TRIB3-F	ACTTTTAAGGAAGCCCGCCGT	94
TRIB3-R	ATTTGCTGGAACAGCCAGGG	
DUSP6-F	GGGTGGATTTGAGGTGCAGT	227
DUSP6-R	GCAGGCGAGACCGAAGTAAA	
IL1β-F	TCCACGTGGGCTGAATAACC	93
IL1β-R	TCGGGCATGGATCAGACAAC	
PTGS2-F	TGATCCCCAGGGCACAAATC	275
PTGS2-R	CAGGAACATGAGGCGGGTAG	
MYC-F	GAAGGGAGATCCGGAGTCAAA	300
MYC-R	CTGCAAGCCCGTATTTCCAC	
ATF4-F	GCACCAAAACCTCGCAACAT	143
ATF4-R	AAGCATCCTCCTTGCTGTTGT	
GAPDH-F	ACCCAGAAGACTGTGGATGG	129
GAPDH-R	CAACAGACACGTTGGGAGTG	

### Western blot assay

*Mycoplasma bovis* P1 and P150 were infected into BoMac cells at an MOI of 1000 and harvested at 6, 12, and 24 h post-infection (pi). Equal numbers of cells were lysed with RIPA lysis buffer with protease inhibitor cocktail and phosphatase inhibitors (Sigma–Aldrich, St. Louis, MO, United States). A BCA protein assay kit (Beyotime) was used to quantify protein concentrations, and 20 μg of total proteins were separated by 12% SDS-PAGE. The proteins in the gel were transferred onto polyvinylidene fluoride membranes (Millipore, Billerica, MA, United States), which were blocked for 1 h at room temperature with 5% (w/v) skim milk in Tris-buffered saline with 0.05% Tween 20 (TBST), and then incubated with different primary antibodies (1:1000 in dilution) overnight at 4°C. The membranes were then incubated and treated with goat anti-mouse IgG or anti-rabbit IgG (1:5000 in dilution, SouthernBiotech) for 1 h after being rinsed three times with TBST. Enhanced chemiluminescence (ECL) reagents (Thermo Fisher, Waltham, MA, United States) were utilized to detect the tagged proteins, with β-actin serving as an internal control. ImageJ software was used to determine the intensity of each band.

### C/EBP homologous protein-specific interference in BoMac cells

Table 2 lists the exact sequences of CHOP gene small interfering RNA (siRNA) oligos and non-target control siRNA (siCtrl) designed and manufactured by Gene Pharma Co., Ltd. (Suzhou, China), which were used to interfere with CHOP gene expression as follows. Then 55 μmol siCHOP was transfected into BoMac cells using jetPRIMER^®^ transfection reagent (Polyplus transfection, French) according to the manufacturer’s manual. The cells were next treated with *M. bovis* P1 and P150 at an MOI of 1,000 for an additional 12 h at 37°C, as described above. After that, the cells were collected for the qRT-PCR and Western blot assay.

**TABLE 2:** Small interfering RNA (siRNA) sequences used in this study.

Name	Sense sequences (5′-3′)	Antisense sequences (5′-3′)
siCHOP-170	GCAGCUGAGUCACUGC CUUTT	AAGGCAGUGACUCAGC UGCTT
siCHOP-514	GCAACGCAUGAAGGAG AAATT	UUUCUCCUUCAUGCGU UGCTT
siCHOP-649	GAUGGUUAAUCUGCAC CAATT	UUGGUGCAGAUUAACC AUCTT
siCtrl	UUCUCCGAACGUGUCA CGUTT	ACGUGACACGUUCGGA GAATT

### Statistical analysis

The statistical analysis was performed with GraphPad Prism 8.0 software (San Diego, CA, United States). The data were expressed as means ± standard deviation (SD) of at least three independent experiments. The significance of the differences in each group was determined with either a two-tailed student’s *t*-test for one comparison or a one-way analysis of variance (ANOVA) for over one comparison. The *p-*values less than 0.05 were regarded as statistically significant difference and denoted in the figures as follows: ns, not significant; *p < 0.05, ^**^p < 0.01, ^***^p < 0.001, ^****^p < 0.0001.

### Data availability statement

Our SRA records were deposited in the NCBI database and will be accessible with the following link: https://www.ncbi.nlm.nih.gov/bioproject/PRJNA769187.

## Results

### P150 induced higher levels of cytotoxicity and apoptosis, but lower intracellular survival

**FIGURE 1:**
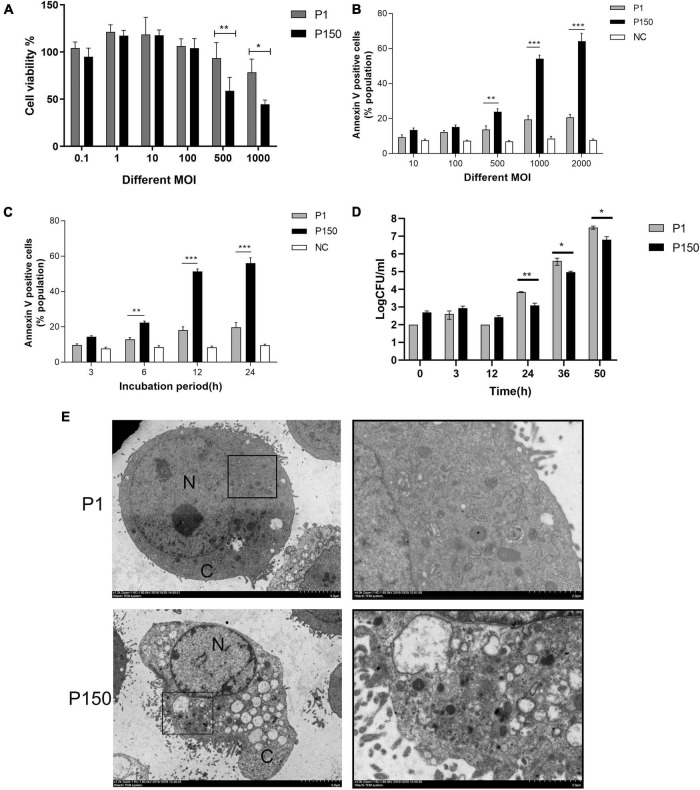
The differential viability and apoptosis of BoMac cells infected by *M. bovis* P1 and P150 and intracellular survival of both strains. **(A)** MTT assay demonstrated that infection decreased cell viability in dose-dependent manner with the high multiplicities of infection (MOI) of 500 and 1000, but P150 showed stronger ability to reduce cell viability than P1. **(B)** Flow cytometry assay on apoptosis of the infected BoMac cells at various MOIs after being stained with Annexin V-FITC and propidium iodide (PI) showed that P150 induced significantly higher levels of apoptosis at high MOIs. **(C)** Flow cytometry assay on apoptosis of infected BoMac cells at various times post-infection at an MOI of 1000 showed that P150 induced significantly higher levels of apoptosis from 6 h onward. **(D)** Intracellular survival and growth of *M. bovis* P1 and P150 in BoMac cells. The data represent the mean ± SD of 3 independent experiments. *, **, and *** represent *p* < 0.05, *p* < 0.01, and *p* < 0.001, respectively. **(E)** TEM micrographs of BoMac cells infected with P1 and P150 where the P150 infected cells are characterized by typical morphology of apoptotic cells including cytoplasmic vacuolation, chromatin condensation and margination, and formation of a crescent. The perinuclear rough ER regions on the images in the left panel are magnified on the right panels. Asterisk indicates ER lamellae infection by P1 and P150. N, nucleus; C, cytoplasm.

An MTT test was used to analyze the cytotoxic effect of *M. bovis* virulent P1 and attenuated P150 strains on BoMac cells. The P150 infection dramatically reduced cell viability at higher MOIs of 500 and 1,000, as seen in Figure 1A. The apoptosis levels of infected BoMac cells were detected using Annexin FITC/PI staining using flow cytometry to further study the reason underlying the lower cell viability. Infection of both strains caused increased levels of apoptosis in a dose- and time-dependent manner when compared to the control group (Figures 1B,C). At high MOIs of 500, 1,000, and 2,000, P150 triggered considerably more apoptosis than P1 (*p* < 0.01) (Figure 1B). The findings showed that both the two *M. bovis* strains increased the proportions of apoptotic cells from 6 h post-infection (pi) onward, compared to the blank control, but the apoptosis levels in the P150-infected cells were significantly higher than those in the P1-infected cells (*p* < 0.01) at 6, 12, and 24 h post-infection (pi) (Figure 1C).

The intracellular survival of mycoplasma was also tested. Although both strains’ intracellular mycoplasma increased with time from 24 h pi onward, more viable bacteria were recovered from P1-infected BoMac cells than P150 (Figure 1D), showing that P1 has a better intracellular survival ability than P150.

After infection for 12 h, the ultrastructure of P1 -and P150-infected BoMac cells was investigated under TEM. The typical morphological traits of apoptotic cells were frequently detected in P150-infected cells, including multiple cytoplasmic vacuoles, chromatin condensation and margination, and the formation of a crescent under the nuclear membrane (Figure 1E). In addition, after *M. bovis* P150 infection, the ER compartment was enlarged—a morphological sign of ER stress. The above morphological change was difficult to discern in P1-infected cells (Figure 1E).

### Differentially expressed genes in BoMac cells induced by P1 and P150

*Mycoplasma bovis* P1- and P150- infected with BoMac cells were collected 12 h to determine the gene expression profile, and six transcript RNA libraries were created, with six samples called P1_1, P1_2, P1_3, P150_1, P150_2, and P150_3. Supplementary Table 1 contains all information on the quality of transcriptomic sequencing. About filtering out the adapter and the valve of Q30, around 27.4–33.6 million clean reads were recovered, and the comparison between the GC content of sequencing results was about 50.08–50.97%, indicating that the sequencing data are suitable for further study. Using TopHat v2.0.12, we mapped the high quantity clean to the cow genome (*Bos taurus*). Total cleaning reads were 95.40–95.67% mapped to the cattle genome, with 93.14–93.44% of them uniquely mapped to a specific location within the cattle genome. Furthermore, the percentage of multi-mapped reads in each sample was less than 5%, which is within industry standards and indicates that these data were available for DEGs analysis.

**FIGURE 2:**
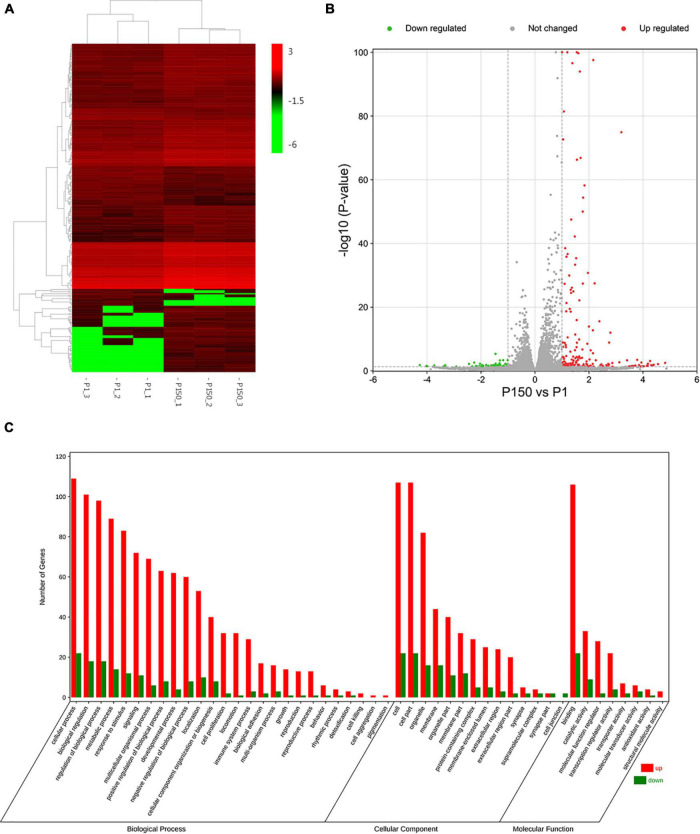
DEGs analysis of BoMac cell after the P1 and P150 infection. **(A)** The heat map showed the expression levels of DEGs in the pair of P150 vs. P1. The red color represents the upregulated expression of genes, while the green color represents a downregulated expression of genes. The gradient color barcode at the right top indicates log_2_(FPKM) value. Each row represents a gene and each column represents a sample. **(B)** Volcano map showed the differential mRNA expression between P150 and P1 (log2FoldChange > 1, *p*-value < 0.05). **(C)** Gene ontology (GO) functional classification of upregulated (Red) and downregulated (Green) DEGs. Genes were annotated in three categories: biological process, molecular function, and cellular component. The *X*-axis means the number of DEGs, while *Y*-axis represents GO terms.

Heat maps were created based on log2Fold change >1 and *p* < 0.05 values to display the findings of clustering analysis using Cluster 3.0 software, in which one little square represents one gene and its color represents the gene’s expression level, as shown in Figure 2A. Each column shows the expression level of all genes in each sample, whereas each row represents the expression level of each gene in a distinct sample. Figure 2B and Supplementary Table 2 show that out of the 16,329 genes in the annotated bovine genome, the clustering analysis revealed 233 DEGs in the P150-infected cells vs. the P1-infected cells, with 185 DEGs upregulated and only 48 DEGs downregulated (log2FoldChange >1, *p*-value < 0.05).

### Gene ontology and Kyoto encyclopedia of genes and genomes pathway enrichment analysis of differentially expressed genes

We used a web-based DAVID database to do GO analysis to learn more about the biological activities of the DEGs. A total of 162 out of 233 profiled DEGs were ascribed to 26 biological processes, 14 cellular components, and 8 molecular function terms. The GO terms of molecular function category were binding, catalytic activity, and molecular function regulator. The top five GO terms in biological functions were mostly related to cellular process, biological regulation, regulation of biological process, metabolic process, and response to stimulus. The top five GO terms in cellular component function included cell, cell part, organelle, membrane, and organelle part. Figure 2C depicts typical GO terms with full data in Supplementary Table 3.

**FIGURE 3:**
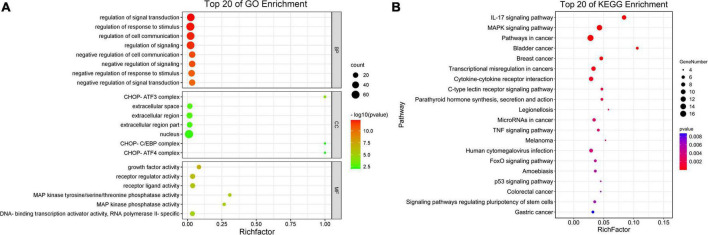
Scatter plot of enriched GO terms and KEGG pathway statistics. Rich factor is the ratio of the differentially expressed gene number to the total gene number in a certain pathway. The color and size of the dots represent the range of the –log10(*p*-value) and the number of DEGs mapped to the indicated terms, respectively. The top 20 enriched GO terms **(A)** and KEGG pathways **(B)** are shown in the figure.

The top 20 enriched GO terms were further examined using the Rich Factor, with the “CHOP-ATF3 complex,” “CHOP-C/EBP complex,” and “CHOP-ATF4 complex” in cellular component function and “MAP kinase phosphatase activity” in the molecular function being the most significantly enriched GO terms (Figure 3A and Supplementary Table 3). Two of the top 20 KEGG enrichment pathways were linked to the immune system, including the IL-17 signaling pathway and C-type lectin receptor signaling pathway. Three pathways were involved in signal transduction, such as MAPK signaling pathway, TNF signaling pathway, and FoxO signaling pathway. The p53 signaling pathway, for example, was implicated in cell growth and death. Three pathways were associated with infectious diseases, such as legionellosis, human cytomegalovirus infection, and amebiasis (Figure 3B and Supplementary Table 4). Based on the *p*-values and GO enrichment score, 20 genes were most significantly upregulated after the P150 infection. Table 3 has further information on these genes. These DEGs potential targets could be important mediators in the *M. bovis* infection.

**TABLE 3:** Mostly upregulated enriched 20 DEGs in *M. bovis* P150 infected BoMac compared to P1 strain.

Gene names	Description	GO terms	log2Fold change	*P*-values
TCIM	transcriptional and immune response regulator	GO:0006915 apoptotic process GO:0045746 negative regulation of Notch signaling pathway	3.20	1.20E-75
HMOX1	heme oxygenase 1	GO:0006915 apoptotic process GO:0043065 positive regulation of apoptotic process GO:0006979 response to oxidative stress	1.21	2.17E-37
CHOP	DDIT3, C/EBP homologous protein	GO:0006915 apoptotic process GO:0070059 intrinsic apoptotic signaling pathway in response to endoplasmic reticulum stress GO:0034976 response to endoplasmic reticulum stress GO:1990622 CHOP-ATF3 complex	1.78	4.22E-55
BNIPL	BCL2 interacting protein like	GO:0006915 apoptotic process GO:0008285 negative regulation of cell proliferation	1.12	0
PPP1R15A	protein phosphatase 1 regulatory subunit 15A	GO:0006915 apoptotic process	1.12	3.16E-39
INHBE	inhibin subunit beta E	GO:0042981 regulation of apoptotic process	1.20	0.01
GDF15	growth differentiation factor 15	GO:0042981 regulation of apoptotic process	1.83	6.26E-59
MYC	MYC proto-oncogene, bHLH transcription factor	GO:0008284 positive regulation of cell proliferation GO:0060070 canonical Wnt signaling pathway GO:0042981 regulation of apoptotic process	1.61	1.89E-100
DUSP1	dual specificity phosphatase 1	GO:0042981 regulation of apoptotic process	1.07	3.70E-82
DUSP6	dual specificity phosphatase 6	GO:0043065 positive regulation of apoptotic process	1.04	2.31E-73
GADD45A	growth arrest and DNA-damage-inducible alpha	GO:0043065 positive regulation of apoptotic process	1.55	1.60E-113
OSGIN1	oxidative stress induced growth inhibitor 1	GO:0043065 positive regulation of apoptotic process	1.27	1.10E-30
TRIB3	tribbles pseudokinase 3	GO:0070059 intrinsic apoptotic signaling pathway in response to endoplasmic reticulum stress GO:0034976 response to endoplasmic reticulum stress	1.77	9.84E-51
CHAC1	ChaC glutathione specific gamma-glutamylcyclotransferase 1	GO:0045746 negative regulation of Notch signaling pathway GO:0070059 intrinsic apoptotic signaling pathway in response to endoplasmic reticulum stress	1.96	1.79E-31
CXCL8	C-X-C motif chemokine ligand 8	GO:0006955 immune response GO:0006954 inflammatory response GO:0070098 chemokine-mediated signaling pathway GO:0034976 response to endoplasmic reticulum stress	2.21	3.24E-28
KLF4	Kruppel like factor 4	GO:0008285 negative regulation of cell proliferation GO:0060070 canonical Wnt signaling pathway	1.10	4.71E-28
SPRY2	sprouty RTK signaling antagonist 2	GO:0008285 negative regulation of cell proliferation GO:0010628 positive regulation of gene expression	1.16	0.000022
CYP27B1	cytochrome P450 family 27 subfamily B member 1	GO:0008285 negative regulation of cell proliferation	1.18	0.004959799
SRXN1	sulfiredoxin 1	GO:0006979 response to oxidative stress	1.49	4.67E-34
PTGS2	prostaglandin-endoperoxide synthase 2	GO:0006979 response to oxidative stress	1.63	0.0000465

log2Fold changes (P150/P1) display the mean value of 3 replicate samples obtained per group. Genes were ranked according to the expression changes detected by RNA-seq.

### Validation of differentially expressed genes involved in inflammatory response and apoptosis

**FIGURE 4:**
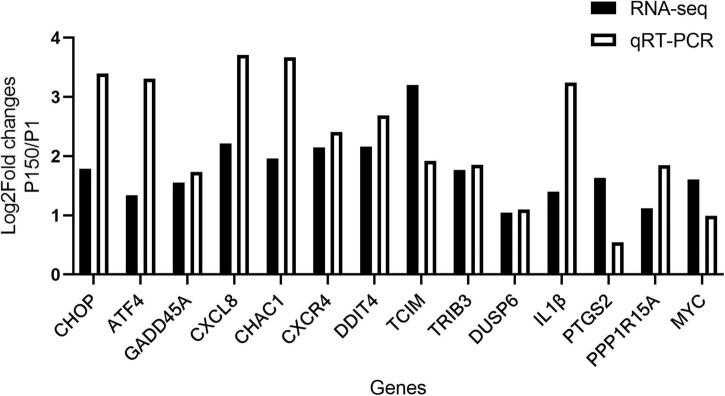
Validation of RNA-seq results by qRT-PCR. Log2Fold changes (P150 vs. P1) obtained by qRT-PCR were compared with the sequencing results for 14 DEGs.

For the qRT-PCR validation, 14 upregulated DEGs implicated in the inflammatory response and CHOP complex were chosen. When compared to P1, the expression of CHOP (DDIT3, DNA damage-inducible transcript 3) and ATF4 (activating transcription factor 4) genes, which are involved in the PERK signaling pathway and the intrinsic apoptotic signaling pathway in response to ER stress, was confirmed to be upregulated in the P150-infected cells. Furthermore, the expression of proapoptotic genes such as CHAC1 (glutathione specific gamma-glutamylcyclotransferase 1), TRIB3 (tribbles pseudokinase 3), GADD45A (growth arrest and DNA damage-inducible alpha), and DUSP6 (dual specificity phosphatase 6) was also upregulated. Furthermore, in P150-infected cells, expression of numerous positive apoptotic genes such as TCIM (transcriptional and immune response regulator), MYC (MYC proto-oncogene), PPP1R15 (protein phosphatase 1 regulatory subunit 15A) was elevated. CXCL8, CXCR4, and IL1B, all of which contribute to the inflammatory response, were also elevated. The relative expression levels of all 14 DEGs were consistent with the RNA-seq results in general (Figure 4). Overall, this verification revealed that infection of BoMac with P150 activated the proapoptotic signaling pathway more than the infection with P1.

### Increased expression of endoplasmic reticulum-stress-associated genes during P150 infection

**FIGURE 5:**
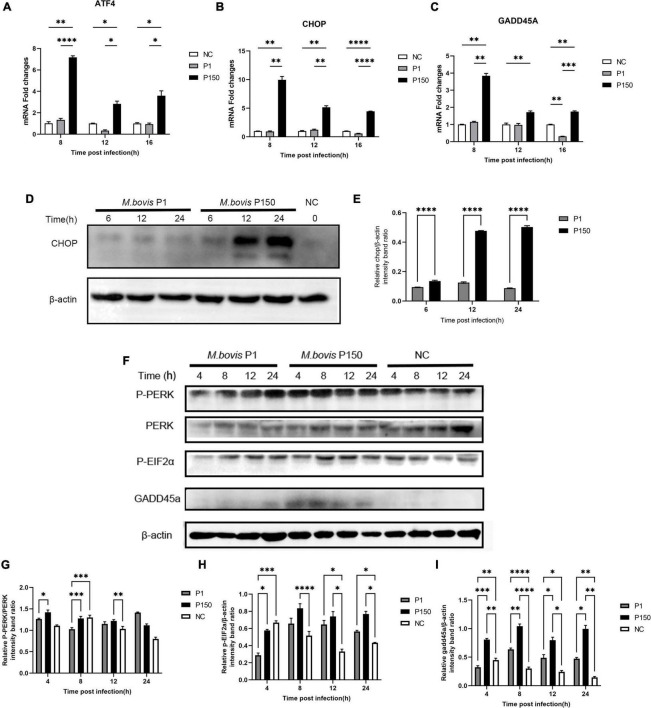
Different expression of ER-stress responsive genes and proteins critical to PERK signal pathway in BoMac cells induced by *M. bovis* P1 and P150 strains. **(A–C)** The mRNA expression of ATF4, CHOP, and GADD45A, respectively, in PERK signal pathway at 8, 12, and 16 h post-infection by *M. bovis* P1 and P150 in BoMac cells using the qRT-PCR assay. Data are presented as the means ± SD of the results from three independent experiments. **(D)** Western blot assay indicated that CHOP was significantly increased in P150-infected cells at 6, 12, and 24 h post-infection compared to P1-infected. **(E)** Represents the relative intensity of western blot bands of panel **D** for expression of CHOP evaluated with ImageJ and normalized to β-actin. **(F)** The effect of *M. bovis* on target proteins in the PERK signal pathway was analyzed by western blot probed with specific antibodies. **(G–I)** Densitometry quantification of P-PERK, P-EIF2α, and GADD45A was calculated by ImageJ analysis. Statistically significant difference was assessed by one-way ANOVA with Dunnett’s multiple-comparison test between P1 and P150 and annotated as follows: *p < 0.05; ^**^p < 0.01; ^***^p < 0.001; and ^****^p < 0.0001.

As shown above, three out of fourteen genes (ATF4, CHOP, and GADD45A) involved in the activation of the PERK pathway during ER stress were upregulated after P150 infection compared to P1. The phosphorylation of the eukaryotic initiation factor 2 alpha subunit (eIF2α) and expression of ATF4, an ER-stress-inducible transcription factor, could be activated by activating the proapoptotic genes CHOP and GADD45A when the PERK pathway is activated (Rozpedek et al., 2016). The P150-infected cells had considerably higher ATF4 mRNA expression at 8, 12, and 16 h pi than P1 and negative control cells, as expected (Figure 5A). In addition, its two downstream genes CHOP and GADD45A were shown to be considerably elevated in P150 (Figures 5B,C). Furthermore, whole-cell lysates were produced and analyzed for 6, 12, and 24 h pi by western blotting. The target protein’s relative levels were normalized to β-actin as an internal loading control protein, and densitometry measurement was determined using ImageJ software. We found that the CHOP protein levels were much greater in the P150-infected cells than in the P1-infected cells at 12 and 24 h, as seen in Figures 5D,E, showing that P150 infection could potentially boost the CHOP expression. We also looked at the expression of PERK, phosphorylated-PERK (P-PERK), phosphorylated EIF2α (P-EIF2α), and GADD45A to see whether the *M. bovis* P150 infection affected the PERK signal pathway. As shown in Figures 5F,G, the levels of P-PERK/PERK were significantly higher in the P150-infected cells than in the P1-infected at 4 and 8 h. The levels of P-EIF2α were both higher in the P1- and P150-infected cells than in the negative control at 8, 12, and 24 h (Figures 5F,H). Interestingly, the levels of GADD45A were much higher in the P150-infected than in P1-infected or negative control cells (Figures 5F,I).

### C/EBP homologous protein knockdown suppressed *M. bovis*-induced apoptosis

**FIGURE 6:**
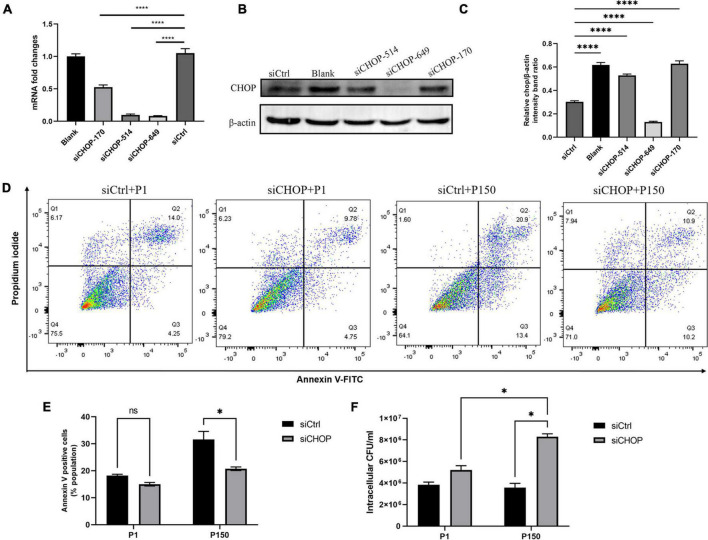
Effect of CHOP knockdown with siRNA interference on apoptosis and intracellular survival of *M. bovis* P1 and P150. qRT-PCR **(A)** and western blot analysis **(B,C)** were performed to evaluate the expression of CHOP in transfected BoMac cells. **(D,E)** Apoptosis assay by flow cytometry showed that CHOP knockdown significantly decreased the apoptosis of BoMac cells induced by P150 infection, but not by P1 infection. **(F)** Quantification of intracellular survival of *M. bovis* P1 and P150 in BoMac cells. Cells were harvested at 24-h post-infection with *M. bovis* and bacteria number was determined by gentamicin invasion assay. Data are presented as the means ± SD of the results from three independent experiments. A significant difference was assessed by one-way ANOVA relative to the control. *p < 0.05 and ^****^p < 0.0001.

Three synthetic siRNAs against CHOP, including siCHOP-170, siCHOP-514, and siCHOP-649, were used to interfere with endogenous CHOP expression to further indicate that CHOP played a critical role during *M. bovis*-induced apoptosis. When compared to the non-targeting siCtrl, all three siCHOP had a significant inhibitory impact (*p* < 0.0001), however, siCHOP-514 and siCHOP-649 primarily decreased the CHOP expression (Figure 6A). Furthermore, the western blot assay confirmed that transfection of BoMac cells with siCHOP-649 almost eliminated the CHOP expression compared to non-targeting siCtrl control (Figures 6B,C). The siCHOP-649 was also used to investigate the effect of CHOP knockdown in apoptosis regulation. Flow cytometry was used to detect apoptosis, and the results revealed that the apoptotic cell percentage (right upper quadrant and right lower quadrant represent the late-stage apoptosis rate and early stage apoptosis rate, respectively) after CHOP knockdown dramatically reduced in the P150-infected cells at 12 h after infection (*p* < 0.001), but did not affect P1-induced cell death (Figures 6D,E).

### Effect of C/EBP homologous protein-mediated apoptosis on intracellular survival of P150

Based on the findings, it is fair to believe that P150-induced apoptosis in ER stress response *via* CHOP could affect intracellular bacteria survival. Before infection with *M. bovis* P1 and P150, BoMac cells were transfected with siCHOP. At 24 h PI. The total number of intracellular bacteria were considerably higher with siCHOP treatment than with siCtrl, and significantly higher than P1 with siCHOP treatment (Figure 6F).

## Discussion

*Mycoplasma bovis* is a significant contributor to the global burden of bovine respiratory illness. In a recent work, an attenuated *M. bovis* strain P150 derived from several *in vitro* passages of the virulent strain P1 elicited a strong innate immune response, suggesting that it could protect calves from infection with the virulent strain P1 (Zhang et al., 2014). However, the pathogenicity of the two *M. bovis* strains to calves differs (Chao et al., 2019). In this investigation, we discovered that the *M. bovis*-attenuated-strain P150 could cause more apoptosis in BoMac cells than strain P1, and we investigated the transcriptional profile of BoMac cells at 12 h during *M. bovis* P1 and P150 infections, respectively. Our findings led to several intriguing conclusions. Validation of 14 elevated genes related to inflammatory response and apoptosis was found by comparing 233 DEGs. CHOP-mediated ER stress was surprisingly enriched as the most notable GO category. We also discovered that the CHOP gene is a key regulator of apoptosis in a PERK-dependent signal pathway and that it may be responsible for the ER-stress-induced apoptosis differences between P1 and P150.

Many studies have been done to develop various types of *M. bovis* vaccines to control its infections, including inactivated vaccines, subunit vaccines, and live-attenuated vaccines. Inactivated vaccines are the most commonly used in studies to prevent infections with *M. bovis* and there are two licensed vaccines for the prevention of *M. bovis* infections in the United States. However, the authors found that the two vaccines were not efficacious in reducing the number of *M. bovis* colonizing and the number of *M. bovis*-specific lesions (Soehnlen et al., 2011). Another attempt was made to develop an effective subunit vaccine against *M. bovis* associated with several immunogenic proteins, such as GAPDH, PdhA, PepA, Tuf, P48, P81, OppA, LppA, PepQ, O256, and DeoB (Prysliak and Perez-Casal, 2016). Unfortunately, there was an insufficient cell-mediated response to the *M. bovis* recall antigens and no protection against the *M. bovis* challenge, although a strong humoral immune response was observed based on the IgG1 and IgG2 serum responses (Prysliak et al., 2013). Whereas live-attenuated vaccines are relatively safe and effective, which have been licensed and accepted by swine, poultry, and cattle producers in many countries (Feng et al., 2013; Zhang et al., 2014; Kanci Condello et al., 2020). However, we still lack information about its mechanism of attenuation and protective immune response. In recent years, apoptosis inhibition by different bacteria has been suggested as a mechanism of survival by allowing the pathogen to replicate and disseminate in the host (Maina et al., 2019). Here, we show the difference in the modulation of apoptosis and survival in BoMac cells by *M. bovis* P1 and P150. The cell viability was dramatically reduced at a high MOI of 1,000. The findings are in partial agreement with previous work that *M. bovis-*induced apoptosis in epithelial cells (MAC-T and EBL cells) *via* a mitochondria-dependent pathway and ER-stress-dependent signaling pathway, respectively (Liu et al., 2020; Wu et al., 2021). Consistent with our previous study, live *M. bovis* P1 induced around 14–15% apoptosis in BoMac cells (Zhao et al., 2021). However, we found that the attenuated strain P150 induced significantly more apoptosis than the wild-type strain P1-infected BoMac cells. Furthermore, we used TEM to examine the morphology of BoMac after infection with *M. bovis* P1 and P150, and discovered obvious vacuolation and enlargement of the ER compartment in P150 infection, indicating that *M. bovis-*attenuated strain exactly induces cell damage and is more serious than P1-infected. Comparative genomics previously showed that one 14.2 kb deleted region covering 14 genes was missing in P150 (Rasheed et al., 2017), but the functions of most deleted genes (9/14) are unknown, and therefore, it is hard to suppose their role in the deleted genes in P150-induced apoptosis. In addition, there are many other kinds of mutations such as 46 SNPs and indels at the nucleic acid level between the P150 and P1, which would be possible to induce cell apoptosis. However, it remains to be verified in the future.

The fact that *M. bovis* P150 infection causes more apoptosis in BoMac cells than *M. bovis* P1 infection could have many ramifications. To begin with, a high amount of apoptosis would help to inhibit bacterial growth in phagocytes by killing pathogens. We also discovered that P150 recovered 24 h after infection and had much fewer intracellular bacteria than P1 after gentamicin treatment, indicating *M. bovis* P150 lacks the ability to survive in BoMac cells compared to strain P1. The findings are in agreement with a study that attenuated *Mycobacterium tuberculosis* H37Ra and *Mycobacterium bovis* BCG strongly induce THP-1 apoptosis than virulent wild-type *M. tuberculosis* H37Rv, which is associated with its reduced intracellular viability (Riendeau and Kornfeld, 2003). Second, apoptosis was previously recognized to destroy germs, avoiding severe host tissue damage and allowing for fast immune system clearance. Furthermore, the higher level of apoptosis generated by P150 could be linked to its increased antigen presentation and immune protection as an attenuated vaccine. *Salmonella enterica* serovar Typhimurium strain VNP 20009 is an attenuated strain with the ability of anti-tumor effects by inducing higher apoptosis and then boosting the innate and adaptive anti-tumor immunity (Li et al., 2020).

The secretory pathway’s key intracellular organelle, the endoplasmic reticulum (ER), is responsible for protein translocation, folding, and post-translational modifications, which allow proteins to be transported to the Golgi and then to vesicles for secretion. Disorder in ER function, often known as “ER stress,” causes death in diverse host cells by initiating the unfolded protein response (UPR) (Jing et al., 2012). There are three branches of UPR that are initiated by distinct ER stress: PKR-like endoplasmic reticulum kinase (PERK), inositol-requiring enzyme 1 (IRE1), and activating transcription factor 6 (ATF6) (Jing et al., 2012). In our research, we discovered that the genes of CHOP, ATF4, and GADD45A in the PERK signal pathway, as well as other proapoptotic genes such CHAC1, TRIB3, DUSP6, TCIM, MYC, and PPP1R15, were increased in P150-infected BoMac cells compared with P1-infected cells. Various intracellular bacteria, such as *Mycobacterium tuberculosis* (Sharma et al., 2021), *Brucella melitensis* (Smith et al., 2013), and *Listeria monocytogenes* (Pillich et al., 2012), have been documented to activate the UPR to control intracellular bacterial number during infection. These findings show that the *M. bovis* P150 infection increases the expression of ER-stress-associated genes in the PERK signal pathway compared to P1 infection, resulting in higher apoptosis than P1 infection. We speculate that the wild-type strain might inhibit apoptosis *via* ER stress. Earlier research has reported that *M. hyopneumoniae* infection inhibited the host UPR by inhibition of all three pathways controlled by PERK, IRE1, and ATF6 (Pan et al., 2020). Although the UPR plays an important function in microbial infectivity, its significance in *M. bovis* pathogenesis is uncertain and requires more research.

C/EBP homologous protein is an important component in ER-stress-induced cell death. By modulating the expression of BCL-2, TRB3, death receptor 5, ERO1α, and PPP1R15A, and perturbing the cellular redox state, CHOP synthesis may trigger cell apoptosis (McCullough et al., 2001; Wang et al., 2009). Furthermore, CHOP increases the expression of GADD45 (growth arrest and DNA-damage-inducible protein), which causes cell apoptosis by stopping protein synthesis altogether (Saha et al., 2010). CHOP is ubiquitously expressed at relatively low levels in typical cells but is substantially expressed in most cells when they are stressed (Chikka et al., 2013). This study looked at CHOP expression during *M. bovis* P1 and P150 infections and found that while *M. bovis* P1 infection reduces CHOP expression, P150 infection might boost it. We anticipated that *M. bovis* virulent strain P1 might interfere with the expression of CHOP protein during the pathogen-induced ER stress because CHOP is the major proapoptotic transcription factor activated by UPR. This could be one of the reasons for the *M. bovis*-infected cells’ delayed apoptosis (Maina et al., 2019). To learn more about the role of CHOP in apoptosis in *M. bovis* P150 infection, we used siRNA against the CHOP gene to reduce apoptosis while also decreasing intracellular survival. These findings imply that *M. bovis* P150-induced apoptosis is mediated by CHOP, a protein that has the ability to effectively govern intracellular survival.

**FIGURE 7:**
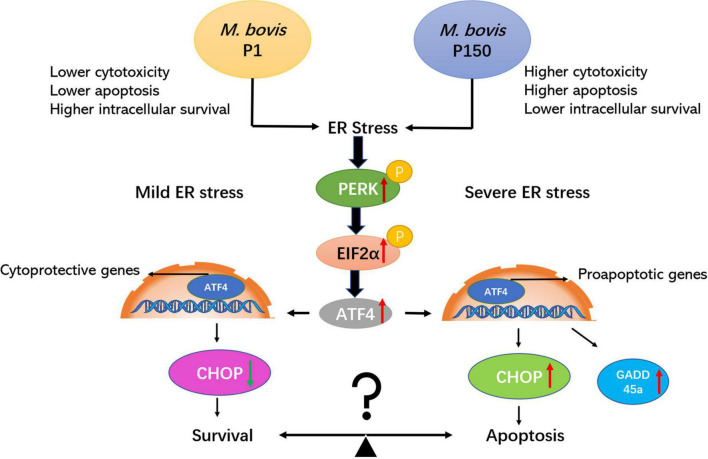
The attenuated *M. bovis* strain P150 induces higher apoptosis of BoMac through a CHOP-upregulated mechanism *via* the protein kinase R-like endoplasmic reticulum kinase (PERK)-dependent signal pathway activation. After *M. bovis* P150 is infected, it induces an ER stress and activates phosphorylation of PERK and subsequently with the ability to phosphorylate EIF2α. The result is the induction of ATF4 mRNA expression, which upregulates the expression of proapoptotic genes, e.g., CHOP and GADD45A. CHOP, the key downstream target of ATF4, as well as GADD45A, are upregulated and subsequently promoted apoptosis. Finally, the apoptosis of cell was increased that might be associated with a lower level of mycoplasma intracellular survival. The PERK/EIF2a/ATF4/CHOP/GADD45A axis is certainly involved in both survival and apoptotic signaling pathways.

As a result of these studies, we propose a possible mechanism in which *M. bovis*-attenuated strain P150 shortens the life of infected bovine alveolar macrophages during ER stress by upregulating the expression of phosphorylated-PERK, phosphorylated-EIF2α, ATF4, CHOP, and GADD45A signaling cascade, thus mediating crosstalk between ER stress and apoptosis signaling (Figure 7). As a result, the balance between apoptosis and survival may be controlled by ER stress caused by *M. bovis* P1 and P150. These findings reveal a variety of possible intervening targets, primarily the apoptotic pathway, and lay the groundwork for more research into the whole or partial apoptosis signaling cascades.

This research has certain drawbacks. First, because this is a transcriptome comparison between *M. bovis* and BoMac, changes in gene expression may not correspond to changes in protein expression as a result of posttranscriptional alteration. Second, increased CHOP was discovered in P150-infected cells, which played a critical role in apoptotic pathway cell fate decisions, however, which key protein prevents CHOP expression from *M. bovis* wild-type strain P1 and whether those missing genes encoded in the 14.2-kb-deleted region affect apoptosis have not been determined. Furthermore, we found that there was no obviously different expression in P-PERK and P-EIF2α during P1 and P150 infection at 12 and 24 h, indicating that the two signal factors were both activated. However, the downstream proteins of CHOP and GADD45A were only upregulated in P150-infected cells. It is still unknown which factor directly induced CHOP expressed *via* the PERK/P-EIF2α/ATF4 axis. Further research is needed to see if there are any additional controlled parameters that can cause host cell survival following infection with the wild-type strain.

## Data availability statement

The data presented in this study are deposited in the NCBI BioProject repository, accession number: PRJNA769187.

## Author contributions

HZ contributed to conceptualization and writing—original draft preparation. HZ and SL contributed to methodology and software. MF, CH, and AG contributed to writing—review and editing. SL and DL contributed to validation. JC and GZ contributed to data curation. MF, YC, and CH contributed to formal analysis. HC, LY, and AG contributed to supervision. AG contributed to project administration and funding acquisition. All authors have read and agreed to the published version of the manuscript.

## Footnotes

1
https://david.ncifcrf.gov/

2
http://kobas.cbi.pku.edu.cn/kobas3/
